# Enlightening dynamic functions in molecular systems by intrinsically chiral light-driven molecular motors

**DOI:** 10.1039/d3cs00247k

**Published:** 2023-08-15

**Authors:** Jinyu Sheng, Daisy R. S. Pooler, Ben L. Feringa

**Affiliations:** a Stratingh Institute for Chemistry, Zernike Institute for Advanced Materials, University of Groningen Nijenborgh 4 9747 AG Groningen The Netherlands b.l.feringa@rug.nl

## Abstract

Chirality is a fundamental property which plays a major role in chemistry, physics, biological systems and materials science. Chiroptical artificial molecular motors (AMMs) are a class of molecules which can convert light energy input into mechanical work, and they hold great potential in the transformation from simple molecules to dynamic systems and responsive materials. Taking distinct advantages of the intrinsic chirality in these structures and the unique opportunity to modulate the chirality on demand, chiral AMMs have been designed for the development of light-responsive dynamic processes including switchable asymmetric catalysis, chiral self-assembly, stereoselective recognition, transmission of chirality, control of spin selectivity and biosystems as well as integration of unidirectional motion with specific mechanical functions. This review focuses on the recently developed strategies for chirality-led applications by the class of intrinsically chiral AMMs. Finally, some limitations in current design and challenges associated with recent systems are discussed and perspectives towards promising candidates for responsive and smart molecular systems and future applications are presented.

## Abstract 2

Chirality is a fundamental property which plays a major role in chemistry, physics, biological systems and materials science.

## Graphical abstract

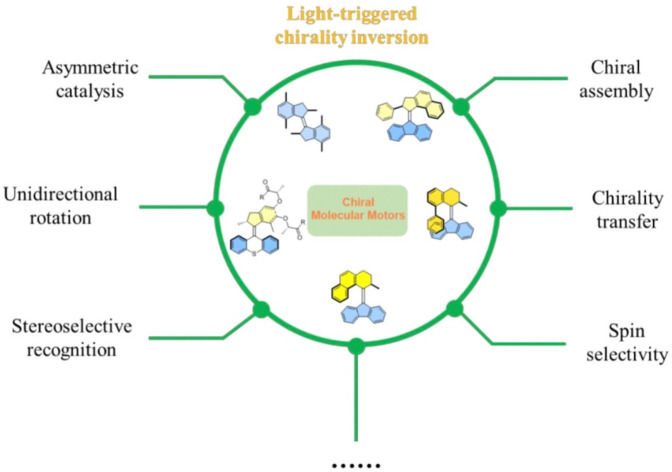

**Key learning points**

1. The importance of dynamic chirality in designing smart materials and molecular systems.

2. The design principles of chiral AMMs for targeting applications.

3. Recent advances using chiral AMMs as multistate chiroptical photoswitches.

4. Representative examples utilizing unidirectional rotary motion of chiral AMMs for various applications.

5. Challenges and prospects for the future of chiral AMMs.

## 1. Introduction

Chirality is a ubiquitous and essential feature in the evolution of Nature, from small chiral molecules to macroscopic objects. In living creatures, molecular asymmetry is commonly regarded as a bio-signature for its central role in biological structures and processes.^1–7^ Apart from the intrinsically chiral small molecules and chiral macromolecular architectures like DNA and proteins, chiral recognition and dynamic transfer of chirality across different length scales in living systems is vital for biological functions,^6–9^ ranging from enzyme catalysis to the helical tendril coiling behavior of cucumbers^10^ and the sensing of dynamic polarization vision by mantis shrimps.^11^ Intrigued by the beauty and prominent role of chirality, chemists have made tremendous efforts in searching for the origin of homochirality in life^1^ – in the context of biogenesis and to elucidate Nature's organizational and molecular recognition principles. In addition, the development of biomedical active compounds based on chiral molecules is of prime importance in pharmaceutical industry and essential in modern society to help to cure patients.^12,13^ Furthermore, the field of asymmetric catalysis, based on metal-chiral ligands^14^ or chiral organo-catalysts^15–19^ and enzyme catalysis^20,21^ is crucial in controlling chirality in synthetic systems ranging from drugs and fragrances to liquid crystal materials.

Despite the major role of small chiral molecules in catalysis and medicinal chemistry, many opportunities offered by the introduction of chirality in materials and molecular systems remained unexplored in the past decades.^4^ This is not surprising, since the chirality of molecules in many applications might have been considered less essential, while synthetic difficulties and challenging chiral separations also likely limited the full exploitation of these molecules in materials science. In recent years the picture has completely changed and novel approaches to control chirality along length scales from molecules and supramolecular assemblies to macromolecular systems enabled unprecedented control over structure and function. In particular as part of the current transition from molecules to dynamic molecular systems, mimicking life-like functions, dynamic control of chirality emerges as a key factor.

**Fig. 1:**
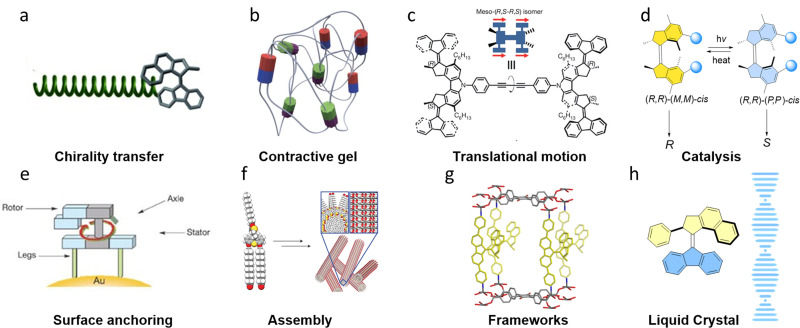
Selected applications of light-driven molecular motors. (a) Chirality transfer from AMM to a dynamic polymer. Adapted with permission from ref. 39. Copyright 2007 Wiley-VCH. (b) AMMs embedded in a contractive gel. Adapted with permission from ref. 41. Copyright 2017, Springer Nature. (c) Electrically driven directional motion of a four-wheeled molecule. Adapted with permission from ref. 44. Copyright 2011, Springer Nature. (d) Enantiodivergent catalysis by AMMs. (e) Surface-assembled unidirectional molecular motor on a gold surface. Adapted with permission from ref. 33. Copyright 2005, Springer Nature. (f) Artificial muscle-like function from hierarchical supramolecular assembly of photoresponsive AMMs. Modified with permission from ref. 42. Copyright 2017, Springer Nature. (g) Embedded AMMs in metal–organic-frameworks. Adapted with permission from ref. 36. Copyright 2019, Springer Nature. (h) AMMs as chiral dopant in liquid crystal materials.^35^

Among the small responsive chiral molecules, chiroptical molecular photoswitches,^22^ especially light-driven artificial molecular motors^23,24^ (AMMs) are appealing due to the intrinsic and dynamic chirality in their structures allowing for the control of chiral functions, as well as driving systems out-of-equilibrium by chirality-controlled unidirectional rotary motion. Due to the non-invasive nature of light used as the stimulus that triggers the rotation of AMMs, these systems are waste-free and allow high spatiotemporal precision; properties that are highly valuable for many applications. Taking these distinct advantages, AMMs have been designed for the development of various light-responsive systems and functions in recent years,^25–29^ including self-assembly,^30–32^ surface modification,^33,34^ liquid crystal (networks),^35^ porous materials,^36–38^ responsive polymers,^39–41^ artificial muscles^42,43^ and autonomous translational motion^44^ (Fig. 1). We would like to emphasize the importance and major potential of dynamic control of chirality in many applications. It is demonstrated that applying intrinsically chiral molecular motors in molecular systems enables unique opportunities. Distinct from chiroptical switches molecular motors featuring unidirectional rotary motion allows to access multiple chiral states in a sequence controlled manner as shown in the photoswitchable asymmetric catalysis and chiral supramolecular self-assembly. A key distinguishing feature is also the directional continuous motion as is evident from the steady state changes of the assembly and chirality of dynamic polymers and rotary and translational movement at the nanoscale. These features are unique to chiral motors enabling to reach out-of-equilibrium states and taking advantage of the chirality to induce continuous directional movement. It should also be noted that: (i) First of all, no external chiral fragment is needed, owing to the intrinsic chirality in the molecular scaffold of the AMM. (ii) Multistate chiroptical control in molecular systems brings a higher level of complexity and sophistication to the dynamic nanomachinery. (iii) Amplification of molecular motion across length scales by overall directional rotation, cannot be realized by either chiroptical photoswitches or racemic molecular motors. Therefore, this tutorial review aims to introduce the importance of various designs and approaches of chiroptical AMMs in chirality-led molecular systems and materials at present, and discuss challenges and future directions.^45,46^

## 2. Light-driven rotary molecular motors

**Fig. 2:**
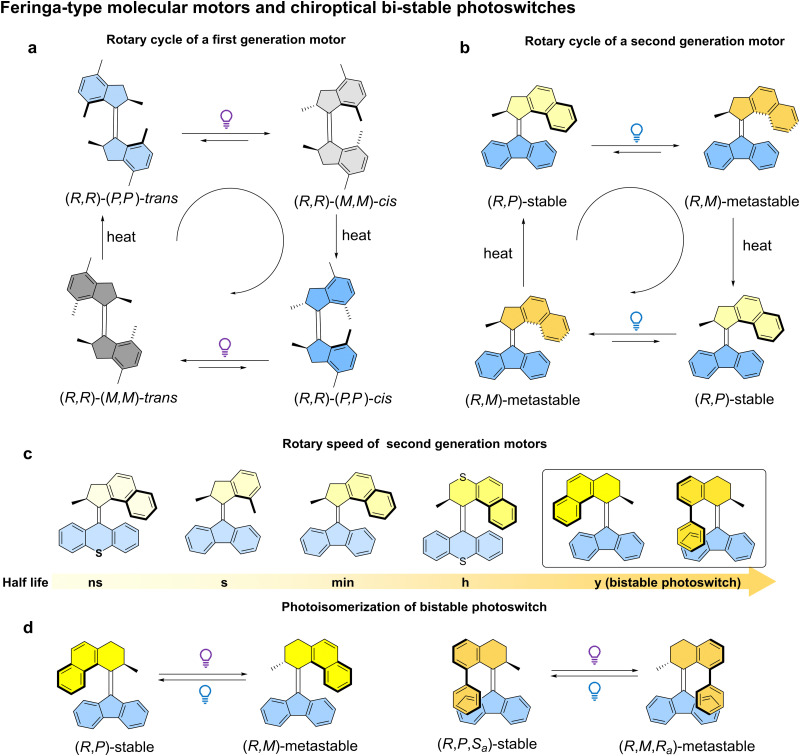
(a) Rotary cycle of a chiral first generation molecular motor. (b) Rotary cycle of a chiral second generation molecular motor. (c) Different half-lives of second generation molecular motors and switches in the ground state at 293.15 K. (d) Photoisomerization of bistable photoswitches with distinct chirality. Only the *R*-enantiomers are shown in this figure.

The first generation molecular motor structure is based on an overcrowded alkene core, with two identical halves on each side of the carbon–carbon double bond (the rotary axle) (Fig. 2a).^23,47^ The steric interactions between the two halves, in the part of the structure which is referred to as the *fjord region*, makes the alkene bond twist out of plane, resulting in the helicity in the structure. Two stereogenic methyl substituents in the structure are preferentially in a pseudo-axial orientation due to steric crowding. These stereocenters dictate the helical chirality in both halves of the molecule, and hence the direction of rotation. Subsequently, second generation molecular motors were developed consisting of two distinct upper (rotor) and bottom (stator) halves (Fig. 2c and d).^48^ In this design, a single stereogenic center was found to be sufficient to induce unidirectional rotation. A full rotary cycle consists of four distinct steps: two photochemical and energetically uphill steps and two thermally activated and energetically downhill steps, *i.e.* thermal helix inversion (THI) steps (Fig. 2a and b). Subsequent studies found the rotary speed could be tuned at will by engineering the degree of steric hindrance at the fjord region.^49^ Generally, upper halves with five-membered rings attached to the alkene bond rotate faster than motors with six-membered ring upper halves, up to a MHz frequency with the design shown in Fig. 2c.^50^ Conversely, the speed could be also slowed down to one cycle in years by engineering a motor core with six-membered upper and five-membered bottom halves. In this case, thermal *E*–*Z* back isomerization is dominated and the metastable isomer generated by light can be triggered by another photochemical stimulus (P-type) to revert to the stable isomer, making it a new class of P-type chiroptical photoswitch (Fig. 2d).^51^

Generally, chiral AMMs can be obtained by chiral separation, either by chiral HPLC or SFC techniques,^52,53^ or through asymmetric synthesis for specific AMM structures.^54–58^ Information regarding the synthesis and purification of chiral molecular motors has been clearly summarized in another recent review, to which we direct the interested reader.^59^ Here, we will display representative examples, focusing on the specific design principles that are required for chirality-led applications. We expand further on some of the areas for which it is evident that chiral AMMs have made an impact in nanotechnology and are promising in the control of various functions.

## 3. Photoswitchable asymmetric catalysis

Photoswitchable catalysis^60,61^ is a rapidly emerging field that shows great potential for non-invasive dynamic control of catalysts, with a switchable feature both towards activity and selectivity. Particularly, the integration of asymmetric catalysis with light-controlled enantioselectivity into one single design is a highly challenging goal. Due to their intrinsic chirality and invertible helical chirality, AMMs and related chiroptical switches^22,62,63^ are of great value as scaffolds in controlling asymmetric catalysis operating as bi- or multi-functional catalysts or ligands.

### 3.1. Molecular motors as multi-state chiral organocatalysts

**Fig. 3:**
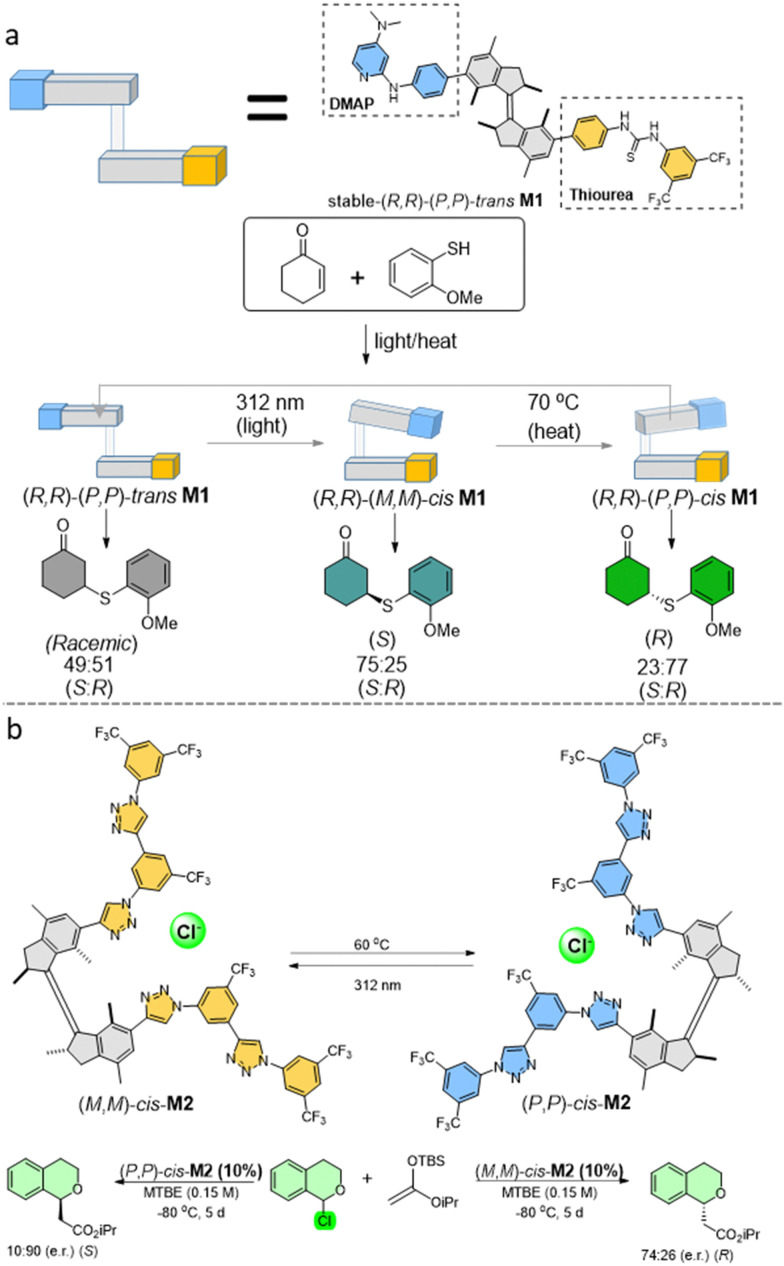
(a) Molecular motor based switchable catalyst M1. Light/heat controlled enantioselectivity in asymmetric catalysis by the different states of molecular motor catalyst. (b) Anion binding asymmetric catalysis based on a first generation molecular motor M2.

Our group developed the first example in which a chiral molecular motor was applied as a catalyst in an asymmetric transformation (Fig. 3a). It could modulate the chirality of the reaction products through the different isomeric states of the motor-based catalyst, which were interconverted by light.^64^ This concept was realized by installing 4-dimethylaminopyridine (DMAP) and thiourea substituents to the motor core as a chiral organocatalyst (Fig. 3a). These two units can cooperate to form a bi-functional chiral organocatalyst, when in close proximity to each other, in the enantioselective addition reaction of thiophenol to cyclohexanone. The strategy involves an activation of the enone through hydrogen bonding with the thiourea and deprotonation of the thiol nucleophile by the DMAP unit. In the enantiopure *E*-isomer of the motor, the lack of cooperative activation led to an inefficient transformation of substrates to a racemic product, showing an “off-state” of our catalyst. On the contrary, two *Z*-isomers with different helicities gave opposite chiral products in a good yield, due to the close cooperation of the functional groups. The stable (*R,R*)-(*P,P*)-*trans* isomer could be isomerized by 312 nm light irradiation to the metastable (*R,R*)-(*M,M*)-*cis* isomer, activating the asymmetric catalysis ability of the motor. Subsequent heating led to the THI step, generating the stable (*R,R*)-(*P,P*)-*cis* isomer, reversing the chirality of the product. Regeneration of the stable (*R,R*)-(*P,P*)-*trans* isomer could be realized by repeating the irradiation/THI steps. Using this light/heat modulation of the motor-based chiral catalyst, the racemic or each enantiomer of the reaction product could be obtained at will. Subsequent studies further proved the elegance of this strategy by dual stereocontrol over the Henry reaction.^65^

Another example showed successful stereodivergent anion binding catalysis with high enantiomeric excess (e.e.) by molecular motors with two oligotriazole anion receptors attached to a first generation motor core (Fig. 3b).^66^ All three motor isomers show 1 : 1 stoichiometric binding affinity with chloride anions in this design, while different isomers show distinct configurations upon anion binding in the formation of supramolecular helical structures. The (*R,R*)-(*P,P*)-*trans* isomer was first investigated as a catalyst in this asymmetric anion binding controlled reaction, with a 1-chloroisochroman derivative as the substrate. The absence of cooperativity between the two oligotriazole branches only gave racemic 1-methoxyisochroman as the product. On the contrary, both *cis* isomers of the catalyst gave the enantiomeric product with different enantiomeric ratio e.r. values. The (*R,R*)-(*M,M*)-*cis* isomer gave lower (e.r.) than (*R,R*)-(*P,P*)-*cis* isomer, possibly due to a less optimal geometry for chloride binding. The Δe.e. value of the products was found to be up to 142%.

### 3.2. Molecular motors as multi-state chiral ligands

**Fig. 4:**
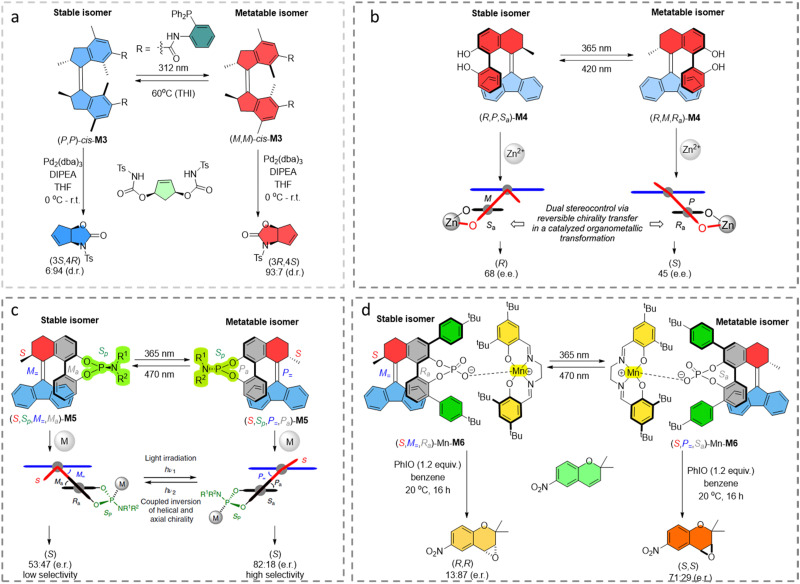
(a) First generation molecular motor based chiral phosphorus ligand for transition metal-based asymmetric catalysis. (*R,R*)-(*P,P*)-*cis*-M3 leads to the product with (3*S*, 4*R*) configuration while (*R,R*)-(*M,M*)-*cis*-M3 gives the opposite (3*R*, 4*S*) chirality. (b) Biphenol based 2nd generation molecular motor M4 as a chiral ligand for asymmetric catalysis. Reproduced with permission from ref. 67. Copyright 2018, American Chemical Society. (c) Phosphoramidite-hybrid 2nd generation molecular motor M5 as a chiral ligand for asymmetric catalysis. Reproduced with permission from ref. 68. Copyright 2020, Springer Nature. (d) Transmission of chirality from molecular motor M6 to achiral manganese(III)-salen catalyst used as a photoswitchable catalyst for asymmetric epoxidation.^69^

Apart from photoswitchable organocatalysts, photoswitchable bidentate phosphorus ligands based on molecular motor cores also proved to be practical in promoting palladium-catalyzed desymmetrization reactions, *i.e.* the asymmetric allylic cyclization of a symmetric bis-carbamate substrate (Fig. 4a).^70^ The (*R,R*)-(*P,P*)-*trans* state gave a racemic product, while the enantioselectivity can be boosted to 93/7 e.r. for (*R,R*)-(*M,M*)-*cis*-state and 6/94 e.r. for the enantiomer product using the (*R,R*)-(*P,P*)-*cis*-state of the catalyst, which is comparable with conventional chiral bis-phosphorous ligands.

By utilizing 2,2′-biphenol-molecular-switch hybrid structures, an overcrowded alkene based bistable chiroptical switch core derivative was used as a photoswitchable catalyst for the enantiodivergent addition of diethylzinc to aldehydes (Fig. 4b).^67^ The selectivity could be tuned by the two different chiral states of the switchable structure, as triggered by two distinct wavelengths of light, 365 nm and 420 nm, respectively. Further transfer of chirality from the catalyst to create another stereogenic element was very successful in the enantioselective addition of diethylzinc to benzaldehyde. The e.e. value of product is up to 68% and the Δe.e. is up to 113% with yields up to 87%, proving the principle of dynamic central-to-helical-to-axial-to-central transfer of chirality.

Phosphoramidite ligands are known as powerful chiral ligands in asymmetric catalysis since being introduced by our group in 1994.^71^ By hybridizing an overcrowded alkene based bistable chiroptical switch core with phosphoramidite ligands, photoresponsive ligands were successfully constructed (Fig. 4c). Taking advantage of the photoswitchable chirality feature of these ligands, this design has been successfully applied in copper(i)-catalyzed conjugate addition of diethylzinc to 2-cyclohexen-1-one to afford an enantiodivergent product controlled by light.^68^

In 2022, the groups of Nolte, Elemans and Feringa developed an elegant photoswitchable chiral anionic ligand, which can axially coordinate and transfer its chirality to an achiral manganese(iii)-salen catalyst (Fig. 4d) as the counterion, making it capable of catalyzing an epoxidation reaction in an enantiodivergent way.^69^ By introducing the phosphoric acid fragment on the upper half of the switch, a hybrid photoswitchable ligand M6 was synthesized. Using an achiral manganese(iii)-salen catalyst, which was known to catalyze the epoxidation reaction of alkenes,^72,73^ the helically chiral photoswitchable ligand can be bound in a supramolecular complex to the achiral manganese(iii)-salen catalyst, inducing chirality in the catalyst. The crystal structures of the metal complex M6 in the stable and metastable states showed the successful chirality transfer from the switch ligand to manganese(iii)-salen catalyst. Studies of the switchable catalytic asymmetric epoxidation showed that the Δe.e. values obtained by this catalyst were up to 118%.

## 4. Switchable and chiral supramolecular self-assembly

Chiral self-assembly processes play an important role in biological systems, such as genetic information transfer and storage, as well as protein folding. The amplification of chirality from the molecular level to the supramolecular level is key in these processes.

**Fig. 5:**
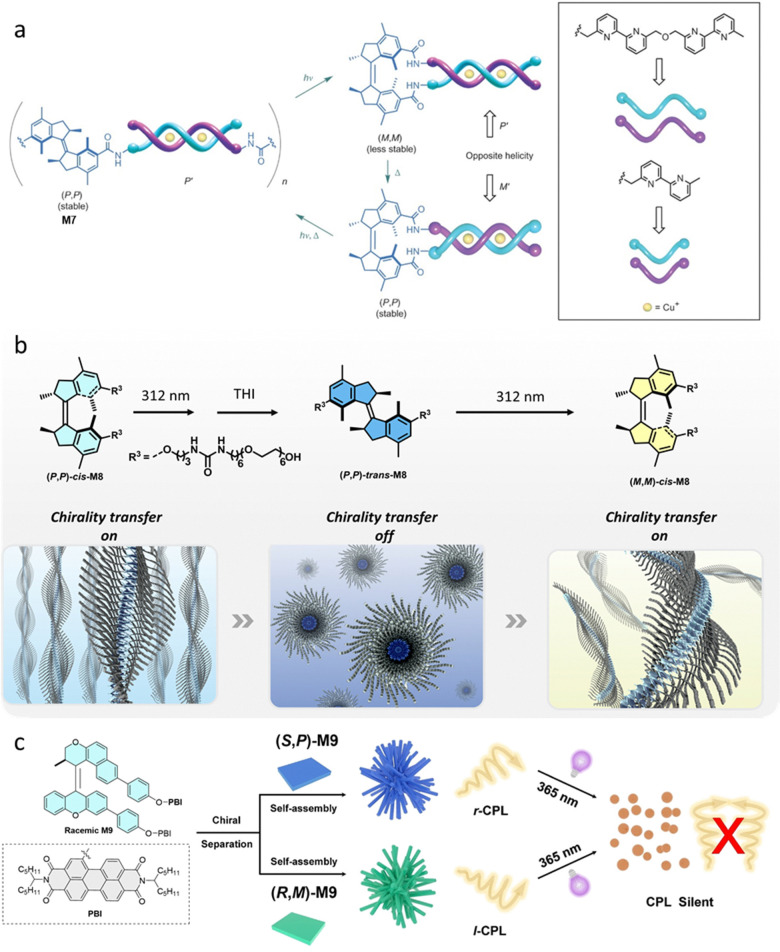
(a) Coordination oligomers formed by (*P*,*P*)-*trans*-M7 with Cu(i), and photoisomerization leads to the formation of P’-helicity of (*M*,*M*)-*cis*-M7 with Cu(i). Thermal helix inversion leads to the formation of M’-helicity of (*P*,*P*)-*cis*-M7 with Cu(i). Modified with permission from ref. 74. Copyright 2017, Springer Nature. (b) Molecular structures of molecular motor M8 in multistate isomers with distinct chirality, and its corresponding assembly structures. Reproduced with permission from ref. 31. Copyright 2022, American Chemical Society. (c) Schematic representation of photoresponsive assembly transformations of enantiopure M9 and photo-controllable CPL signals. Reproduced with permission from ref. 75. Copyright 2022, Wiley-VCH.

Inspired by the supramolecular double helicate structures developed by Lehn and coworkers,^76,77^ our group showed that by functionalizing a chiral first generation molecular motor with two oligobipyridyl ligands, the system is capable of coordinating with copper ions into double-stranded helicates (Fig. 5a).^74^ The molecular motor in the *trans* state with copper ions forms oligomeric structures by intermolecular coordination while the two *cis* states form opposite chiral double-stranded helicates. This system successfully transfered chirality from the motor core to the chiral assembly of the double-stranded helicates, and allows the assembly and interconversion of chirality in a non-equilibrium supramolecular system.

Recently, a multi-state photo-responsive supramolecular polymer based on a homochiral first generation molecular motor was developed, that could transfer chirality from molecular level to various supramolecular architectures through self-assembly in water.^31^ The morphology changes of the supramolecular polymers showed by light/heat control, on–off chirality transfer could be achieved (Fig. 5b). C_6_ alkyl-linkers were positioned between the hexaethylene glycol chains and the urea groups to provide a hydrophobic pocket which facilitated H-bonding, thus enabling the supramolecular assembly. The racemic stable-*cis*-M8 structure in water forms fibers, while by introducing homochirality in (*P*,*P*)-*cis*-M8, a helical structure of the supramolecular polymer is formed. Micelles are formed in the (*P*,*P*)-*trans*-M8 state by 312 nm light irradiation of (*P*,*P*)-*cis*-M8 with a subsequent THI step. Irradiation of (*P*,*P*)-*trans*-M8 sample at 312 nm, (*M*,*M*)-*cis*-M8 and (*P*,*P*)-*trans*-M8 at photostationary state (PSS) in a ratio of 32 : 68 exhibited worm-like fibers. The subsequent THI step did not regenerate the initial morphology, possibly due to the low ratio of (*P*,*P*)-*cis*-M8 in the mixture. Hence, a H_2_O/THF (7/3) solvent mixture was applied to increase the PSS to 70 : 30, which allowed the THI step to recover the initial morphology.

In another application from Qu and co-workers it was shown that a chiral overcrowded alkene unit bearing two PBI units could self-assemble in a chiral manner in its stable state, and exhibits circularly polarized luminescence (CPL).^75^ In this study, the l stable isomer self-assembles into well-defined nanofiber-based clusters in methylcyclohexane, due to the strong π–π stacking interactions of PBI units (Fig. 5c). This chiral self-assembly structure of PBI-AMM (*S*,*P*) shows a right-handed CPL signal (R-CPL) with a *g*_lum_ of 5.4 × 10^-3^ in solution (Fig. 5c). Under 365 nm light irradiation, the stable isomer is converted into the corresponding metastable isomer, causing the assembly morphology of PBI-AMM to transform into discrete nanospheres. This occurs concomitantly with a gradual disappearance of the CPL signal (*g*_lum_ = 0), possibly due to destruction of the co-facial π–π stacking interactions. This example further confirmed the ability of chiral AMMs to transfer their chirality along different length-scales using a dynamic self-assembly strategy.

## 5. Stereoselective guest binding/recognition

Controlling molecular recognition is an important and central goal of the field of supramolecular chemistry. In particular, chiral recognition, such as enantioselective discrimination, is extremely appealing due to its significance in manufacturing drugs and controlling biological processes. Applying external stimuli, especially the use of light-triggered switches, capable of dynamic control over binding properties of artificial receptors in a harmless manner has proven to be a highly promising strategy towards achieving this goal. In particular, intrinsically chiral AMMs are privileged candidates among most of the other switches designed for manipulation molecular chiral recognition.

**Fig. 6:**
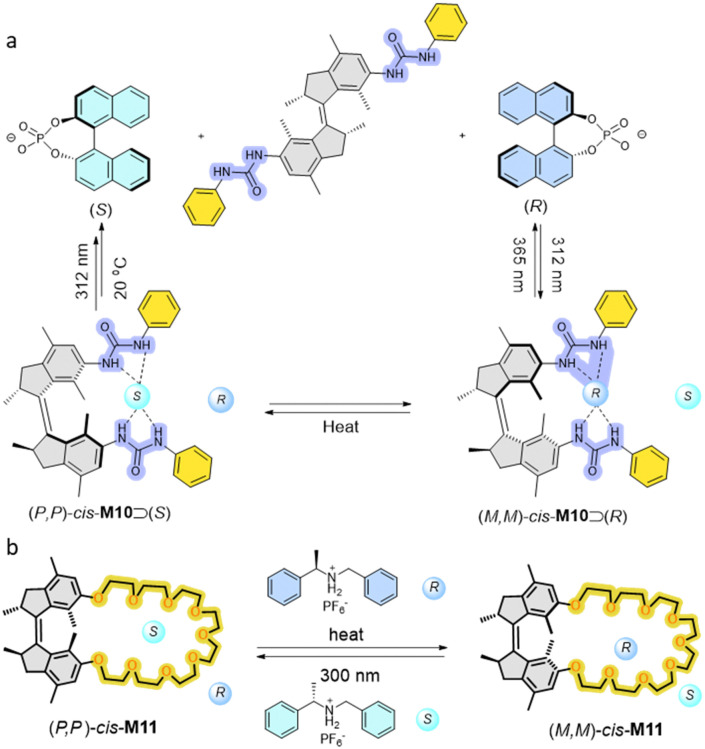
(a) Isomerization of M10 and selective chiral guest recognition of M10 in the (*M*,*M*)-*cis*-form and (*P*,*P*)-*cis*-form. (b) Representation of stereoselective guest recognition of molecular motor-based crown ether M11.

Enantiopure first generation motors functionalized with two urea moieties can serve as chiral receptors to enantioselectively bind to a chiral Binol phosphate anion guest (Fig. 6a).^78^ Both (*P*,*P*)-*cis*-M10 and (*M*,*M*)-*cis*-M10 fit the 1 : 1 binding model towards a chiral phosphate. (*P*,*P*)-*cis*-M10 preferentially binds to (*R*)-phosphate with a *K*_*R*_/*K*_*S*_ = 4.2 ratio. On the contrary, (*M*,*M*)-*cis*-M10 shows an opposite bias as a *K*_*S*_*/K*_*R*_ = 3.2 ratio was obtained. This pioneering result shows that an artificial functionalized motor, here a chiral AMM, could serve as a receptor capable of enantiomer recognition in a dynamic fashion.

Another stereoselective guest recognition motor was based on a motorized macrocycle host–guest system (Fig. 6b).^79^ By tuning the length of oligo-ethylene glycol chains attached to the first generation motor core, unidirectional rotation of the motor host was accomplished. Subsequently, enantiomerically pure motorized macrocycle M11 with a crown ether functionality was separated by chiral HPLC, and was subsequently used to establish dynamic control over binding affinity of a chiral dialkylammonium guest in a 1 : 1 binding model. Preferential binding was found in this host–guest system, *e.g.*, (*P*,*P*)-*cis*-M11 exhibited selectivity for binding of the (*S*)-enantiomer of the ammonium salt (*K*_*S*_/*K*_*R*_ = 1.7). By contrast, (*P*,*P*)-*trans*-M11 shows a poor binding affinity to the guest as the crown ether moiety is less accessible. Subsequent photoisomerization of motorized macrocycle to a (*P*,*P*)-*trans* and (*M*,*M*)-*cis* mixture led to the reversed binding bias, *i.e.*, the (*R*)-enantiomer of the ammonium salt binds stronger with a ratio of binding constant *K*_*R*_/*K*_*S*_ = 2.5.

## 6. Chirality transfer approaches from AMMs

The transmission of chirality through space and across length scales by AMMs can enable dynamic control of function for diverse applications in various areas in chemistry, biology and materials science.^26,28,80,81^ Enantiomerically pure AMMs compounds are prerequisite in order to dynamically deliver and amplify chirality from molecular to macromolecular or supramolecular level.

### 6.1. Switching the helicity of polymers

**Fig. 7:**
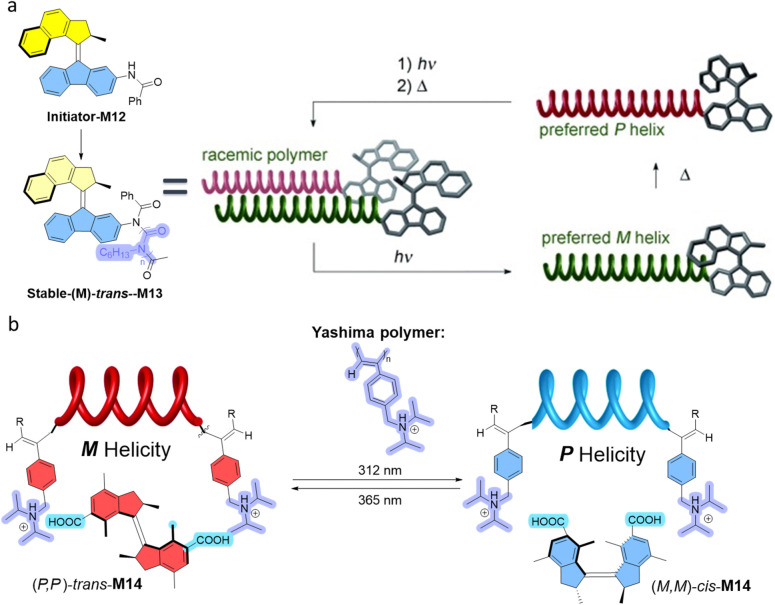
(a) M12 is the initiator to form the poly-isocyanate, whose chains fold into helices. The (*M*)-*trans*-M13 shows little influence on the helical poly-isocyanate, thus the polymer helices are racemic. (*P*)-*cis*-M13 leads to *M*-helical polymer. (*M*)-*cis*-M13 results in a *P*-helix polymer. Reproduced with permission from ref. 39. Copyright 2007, Wiley-VCH. (b) Control over the helicity of polyphenylacetylene using M14 as dopant. Reproduced with permission from ref. 40. Copyright 2017, the Royal Society of Chemistry.

Our group employed an optically pure molecular motor as the initiator for the polymerization of isocyanates (Fig. 7a).^39^ Polyisocyanate is a special dynamic polymer that can fold its chain into helices in response to chiral information.^82^ Thus, the use of a chiral molecular motor in its different isomeric states could control the helicity of the resultant isocyanate polymers. In the stable-*trans* state the obtained polymer is racemic. Subsequent irradiation of racemic motorized polymer, leads to the exclusive formation of the *M*-helical polymer as a result of chirality transfer. After thermal helix inversion, the stable-*cis* motorized polymer exhibits *P*-helicity, reversing the helicity as a result of the chirality transfer process from molecular motor to dynamic isocyanate polymer.

Another example pertains to the chirality transfer from a molecular motor to a dynamic helical polymer, the so-called Yashima polymer, *via* non-covalent ionic interactions (Fig. 7b).^40^ The Yashima polymer consists of water-soluble polyphenylacetylene with ammonium side groups, which enables ionic interaction with a dicarboxylic acid functionalized motor M14. The polymer was saturated at a molar ratio of about 0.025 : 1 (motor : monomer unit in polymer), which represents an efficient transfer of chirality from the dopant to the polymer. Photoisomerization of motor-doped polymer in this case was achieved by first disassociation, then re-association with the polymer after isomerization of the motor to induce the opposite chirality. This *in situ* light irradiation inverts the chirality of motor, which then in turn is transferred to dynamic helical polymers, establishing control of the polymer helicity.

### 6.2. Amplification of chirality in a liquid crystal (network)

Liquid crystals (LCs) with long-range organization and fluidity show great potential for chiral transmission and amplification, and high responsiveness to small chirality changes at the nanoscale.^83^ Molecular motors show change in helicity upon irradiation accompanied by large changes in helical twisting power (HTP), leading to applications in motor-doped systems such as rotating objects on a surface,^35^ supramolecular vortices,^84^ swimming^85^ and reconfigurable chiral droplets,^86^ as well as adaptive optical materials.^87,88^ A recent review by Yang and co-workers discusses the relationship between AMMs and liquid crystals in detail.^89^ Therefore, here, we select a few representative examples to show the interaction between optically pure AMMs as multi-state photoswitches and liquid crystal phases (LC network).

**Fig. 8:**
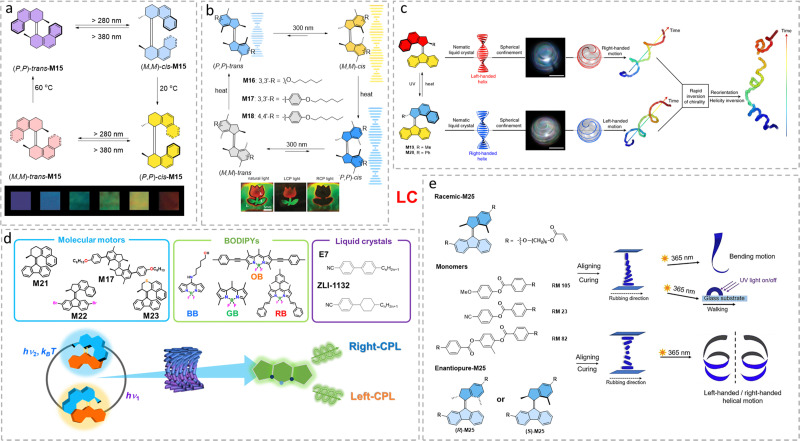
(a) Four-step unidirectional rotation cycle of molecular motor M15. Color changes from left to right correspond to 0, 10, 20, 30, 40, and 80 s of irradiation time, respectively. Adapted with permission from ref. 90. Copyright 2002, the National Academy of Sciences. (b) Four steps rotary cycle of “first generation” AMMs used in this study. The thin cholesteric layers with recorded patterns were visualized under natural (nonpolarized) light and under left- and right-circular polarized light (LCP and RCP, respectively). Adapted with permission from ref. 91. Copyright 2020, Wiley-VCH. (c) Spiral droplets reorient their swimming direction in response to light-induced helix inversion. Reproduced with permission from ref. 85. Copyright 2019, Springer Nature. (d) Molecular-machine-based CPL-emitting LC devices are prepared by mixing AMMs and BODIPYs in different LC mixtures. Reproduced with permission from ref. 93. Copyright 2022, Wiley-VCH. (e) Molecular rotary motor-based photo-responsive liquid-crystal network (LCN) and structure of the LC monomers and molecular motors as cross-linkers. The obtained ribbon is able to bend upon UV light irradiation (365 nm) or walk over a surface (right top). LC monomers with enantiomerically pure motors (*R*)-M24 and (*S*)-M24. The resulting ribbon with R-motor shows left-handed helical motion upon UV light irradiation while the ribbon with S-motor shows right-handed helical motion (right bottom). Reproduced with permission from ref. 94. Copyright 2022, Wiley-VCH.

Doping optically pure first generation molecular motor (*P*,*P*)-trans-M15, into nematic LC (E7) with a weight ratio of 6.6% enables a violet colored reflection band to be observed.^90^ Irradiation with UV light led to the isomerization of (*P*,*P*)-trans-M15 (Fig. 8a), resulting in the helical twisted pitch (HTP) decrease of M15 in a LC film. As a result, clear reflection color changes from violet to red can be observed (Fig. 8a). The removal of UV light gradually led to the regeneration of (*P*,*P*)-trans-M15 thermally by THI, thus recovering the initial reflection band. In this case, the reflection wavelength can be tuned readily throughout the entire visible spectrum simply by changing the irradiation time.

Manipulation of multistate LC phases by first generation motors has been subsequently achieved.^91^ The motor undergoes unidirectional rotation *via* four steps by light/heat manipulation (Fig. 8b). Owing to the short half-life time, here the (*M*,*M*)-*trans* isomer is quickly converted to (*P*,*P*)-*trans* isomer. On the contrary, the (*M*,*M*)-*cis* isomer exhibits a longer half-life, up to 25 d. The (*M*,*M*)-*cis* isomer can be either converted to (*P*,*P*)-*cis* isomer by a THI step or (*P*,*P*)-*trans* isomer by irradiation at 365 nm, thus enables a three-state manipulation of this system. By modifying molecular motor structures (Fig. 8b), the changes in HTP_wt%_ shows the efficient and tunable chirality transfer from molecular motors to liquid crystal phase. By selective UV light exposure to specific areas with different time scales, demonstration of multiple patterning demonstrations has been achieved (Fig. 8b).

Katsonis and co-workers developed a motile LC droplet system that shows light-responsive behavior.^85^ The helical motion of LC droplet is controlled by the helix of the LC phase. The handedness of the trajectory is constant over time, and is always opposite to that of the liquid crystal, *i.e.*, a left-handed helix of the LC gives right-handed motion of the droplet (and *vice versa*). The key to the helical motion of the LC droplet is the Marangoni effect.^92^ That is, the presence of surfactants in water can form a micellar morphology, which solubilizes small amounts of the LCs, creating inhomogeneities in surface tension that are compensated by a flow that drives the droplets forward. Doping a chiral second generation molecular motor (M19 or M20) into this system transfers its chirality to modulate the helicity of LC (Fig. 8c). Subsequent illumination leads to rapid helix inversion (*ca.* 1 s for complete inversion of helicity) of the molecular motor, which thus reorients the direction of the moving droplet.

Programming CPL with a high degree of circular polarization (*g*_lum_) in a dynamic and reversible way *via* external stimuli is important and appealing for its application in optoelectronic devices, including externally addressable materials for displays. Recently, an example shows that doped chiral molecular motors and achiral dyes in LC phase can achieve dynamic control of CPL by chirality transfer from molecular motors to achiral BODIPY dyes (Fig. 8d).^93^ The helicity of the molecular motors can be effectively transferred into LC phases to modulate the chirality of LC phase between left- or right-handed, through light irradiation. Meanwhile, achiral BODIPY dyes dispersed in the LC host could emit a strong CPL signal, which indicated the successful chirality transfer to this fluorescent dye. The remarkable high QY_PL_ (up to 0.75) and *g*_lum_ (up to 0.45) showed the powerful interplay in this design, together with the reversibility and non-invasiveness of this system. By doping different molecular motor cores (M17 and M21 to M23) and dyes (OB, BB, GB and RB), the emission color (from blue to red) can be programmed with different states of chiral molecular motors.

Instead of doping chiral molecular motors in LC phases, integrating chiral molecular motors as cross-linker units into liquid-crystal networks (LCNs) holds promise to not only maintain functions as dopants in LCs, but also results in more complex functions which could be utilized in applications such as soft robotics. To control sophisticated motion by molecular motors, our group successfully copolymerized a second generation molecular motor functionalized with diacrylate into a LCN by a polymerization process.^94^ Interestingly, when irradiating the motor-doped LCN with UV light, the racemic motor M24 led to simple bending motion, while the enantiopure motor resulted in helical motion of the ribbon *i.e.*, left-handed helical motion for (*R*)-M24 and right-handed helical motion for (*S*)-M24 (Fig. 8e). In a subsequent study this concept was applied to realize programmable complex motion in LCNs such as wavy and complex helical motions, which depend on the different chirality of molecular motor units in the LCNs.^95^

## 7. Switchable spin-selectivity

**Fig. 9:**
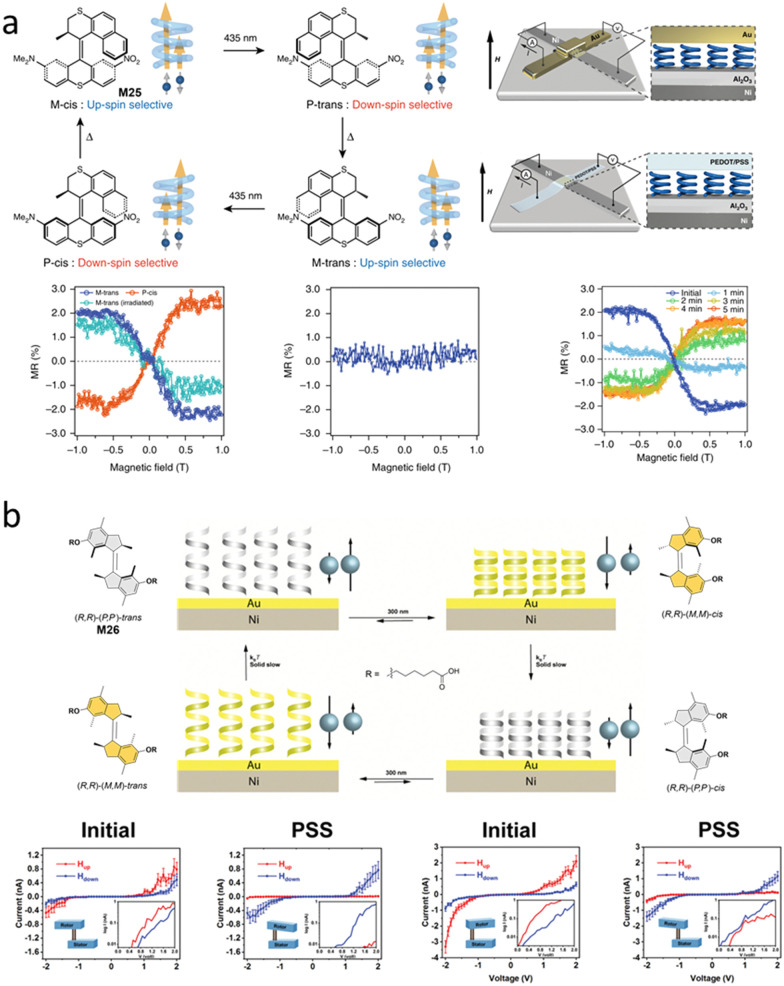
(a) Schematic depiction of the four-stage spin polarization switching in electron tunneling through the molecular motor M25 thin film on the Ni (50 nm)-Al_2_O_3_ (3 nm)-M25 (2–3 nm)-Au (20 nm) or the Ni (50 nm)- Al_2_O_3_ (3 nm)-M25 (2–3 nm)-PEDOT/PSS (600 nm) cross-bar tunnel junction device. Blue helices represent M25. Reproduced with permission from ref. 98. Copyright 2019, Springer Nature. (b) Schematic depiction of the four-stage spin polarization switching in electron tunneling through the molecular motor M26 thin film on nickel/gold (Ni/Au) substrate which is corresponding to unidirectional rotation through four helical states. Reproduced with permission from ref. 100. Copyright 2021, Wiley-VCH.

Spin-selectivity induced by chiral molecules shows great potential to construct and develop spin filters.^96,97^ To control dynamic control of spin-selectivity enabled by chiral switchable AMMs, in 2019, the first example was reported by sandwiching a thin layer of second-generation AMM core M25 between Al_2_O_3_ (3 nm)/Ni (50 nm) and a gold (20 nm) electrode (Fig. 9a) or a poly(3,4-ethylenedioxythiophene, PEDOT): poly(styrenesulfonate, PSySf) (600 nm) electrode (Fig. 9a).^98^ A donor–acceptor-type second generation molecular motor M25 was used in this work to ensure visible-light operation of the system.^99^ Interestingly, in both electrodes, spin-valve-like measurements displayed a clear up-spin selectivity for the *M-trans* device and down-spin selectivity for the *P-cis* device (Fig. 9a). As a control device with racemic M25, no magnetoresistance (MR) signal was found (Fig. 9a). However, the rigidity using a gold electrode system made that the inversion of chirality of M25 was constrained while using a flexible (PEDOT/PSySf) electrode, the inversion of spin selectivity was successfully achieved (Fig. 9a). This result proves the concept that the chiral AMMs could be used to switch the spin-selectivity by inversion of its chirality in solid-state devices.

To achieve multi-state spin selectivity control, a first generation molecular motor M26 (Fig. 9b) based system was developed to switch selectivity *via* the four states of chirality inversion of the motor.^100^ The motor M26 was drop-casted on a gold substrate. In the initial (*P*,*P*)-*trans*-M26, the averaged current magnitude measured with the magnetic field pointing up (*H*_up_) is higher than for magnetic field pointing down (*H*_down_) for all non-zero voltages (Fig. 9b). UV light irradiation at PSS led to reversal in the observed spin selectivity, *i.e.*, higher current for *H*_up_, due to the opposite chirality of (*M*,*M*)-*cis*-M26 isomer (Fig. 9b). Thermal relaxation over days led to the reversed spin selectivity to its original preference, in accordance with the formation of (*P*,*P*)-*cis*-M26. Starting from (*P*,*P*)-*cis*-M26 in an identical study gave consistent results though formation of (*M*,*M*)-*trans*-M26, (Fig. 9b), followed by THI to (*P*,*P*)-*trans*-M26. Interestingly, the motor rotation is uncompromised on the gold surface, but the barriers of the THI steps are quite different compared to those in solution. A drop-casted sample on the surface showed similar thermal half-lives of the thermal helix inversion steps of both metastable *cis*-M26 and metastable *trans*-M26 isomers (*ca. t*_1/2_ ≈1 h). In THF, the half-lives were determined to be *t*_1/2_ =21 h and *t*_1/2_ =10 s, for metastable *cis*-M26 to stable *cis*-M26 and metastable *trans*-M26 to stable *trans*-M26, respectively.

## 8. Continuous unidirectional rotation based applications

In the previous sections, we have described multiple cases in which the chirality of the different isomers of light-driven molecular motors are used for their applications. Additionally, AMMs are also unique for their unidirectional rotational motion controlled by the chirality of the substituent at the stereogenic centre (*R* or *S*) at the allylic position of the overcrowded alkene (resulting in clockwise and anti-clockwise rotation). Thus, targeting applications where uniform directionality is paramount, the molecular motors integrated into the system must be completely homochiral in order to amplify the unidirectional motion, instead of cancelling it out – which would occur in the case of a racemate. Here we describe a few examples that exploit enantiomerically pure molecular motors to amplify molecular motion across length scales.

### 8.1. Rotating an object and dynamic supramolecular chiral structures in LC

**Fig. 10:**
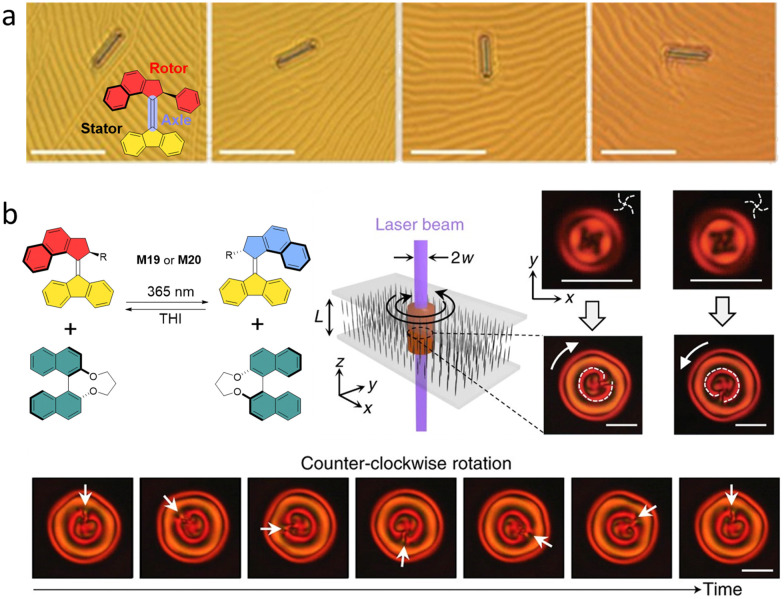
(a) Rotation of the cholesteric LC texture and glass rod by M20. Scale bar: 50 μm. Reproduced with permission from ref. 84. Copyright 2006, Springer Nature. (b) Molecular motor M19 or M20 used as dopant to revolve supramolecular chiral structures in LC materials.

A second-generation motor M20 was used as a chiral dopant in a cholesteric LC film to rearrange the organization in the mesogenic phase by light-induced helicity changes of motor. Remarkably, the change in chirality due to rotation of M20 resulted in autonomous rotation of an object whose size is 10 000 times larger than the molecule itself.^35^ Triggered by 365 nm light, the rotation of the motor changes the surface texture and induces the unidirectional rotation of the rod object (Fig. 10a). When the PSS reached, the rod stopped rotating. The subsequent THI step changes the helicity of motor, leading to the opposite rotation direction of the object.

Brasselet, Katsonis and co-workers used AMMs as dopants and binaphthyl as a co-dopant to revolve supramolecular chiral structures in LC materials continuously.^84^ Here the chiral co-dopant is essential to pre-program the direction of rotation at the supramolecular level (Fig. 10b). The rotation at the supramolecular level is sustained by the diffusion of the motors away from a localized illumination area. The diffusion is controlled by the power of the illuminating beam, since the twisted LC structure stays stable under low power of the beam (Fig. 10b). Here, the chiral molecular motor M20 was used mainly for continuously rotating through the strong light illumination that drives the system out of equilibrium by the disturbance of helical structure. This was further confirmed by using opposite chirality of M20 in this system, which led to the same behavior. Above a critical irradiation power, by diffusion of the AMM away from the illuminated area, a spontaneous symmetry breaking dictates the directionality of the supramolecular rotation (Fig. 10b). Remarkably, this system is proven to be extremely robust, even after aging for three years.

### 8.2. Molecular nanocar

**Fig. 11:**
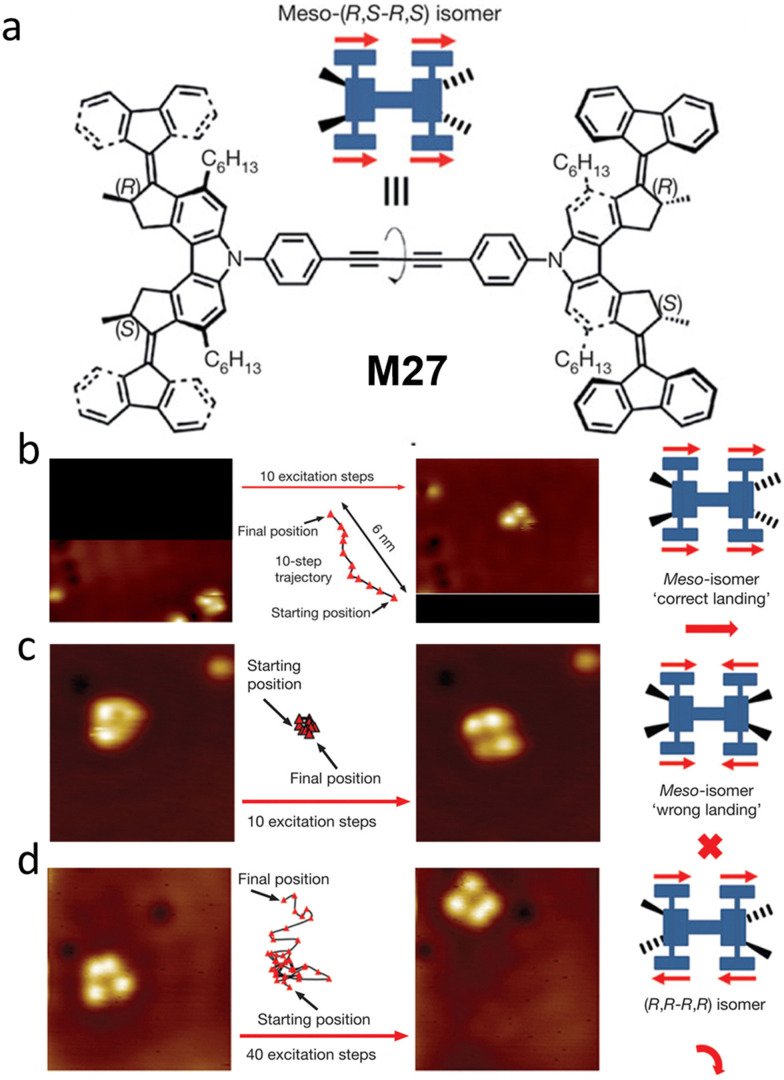
(a) Structure and cartoon representation of the meso-(*R*,*S-R*,*S*) isomer M27. (b)–(d). STM images of molecular nanocars’ movements with different isomers. Reproduced with permission from ref. 44. Copyright 2011, Springer Nature.

Our group designed a molecular “nanocar” structure M27 with four motor units integrated into a single molecule (Fig. 11a).^44^ In this design, the four-wheeled molecule could “walk” on a Cu (111) surface by the cooperative rotation of the AMM units upon sequential electronic and vibrational excitation. By tuning the chirality of the individual motor units, the molecule can be adapted to different trajectories on surface. Interestingly, when the *meso*-(*R*,*S-R*,*S*) isomer is adsorbed onto the surface in the proper orientation (“correct landing”, Fig. 11b), the four motor units can behave in the correct way to move the molecule translationally along the surface up to 6 nm *via* a series of conrotatory motions. In contrast, the “wrongly landing” *meso*-(*R*,*S-R*,*S*) isomer cannot move because of the combined “cancelling out” effects of the motor units, thus impeding translational movement of the molecule (Fig. 11c). The (*R*,*R-R*,*R*) and (*S*,*S-S*,*S*) isomers rotate in a disrotatory fashion, resulting in random movements or ideally spinning motion (Fig. 11d). This exquisite work unequivocally revealed the great importance of chirality in molecular design to achieve directional movement, and showcased the beauty of combining multiple chiral units resulting in the conversion of rotational movement to translational movement.

### 8.3. Contraction of hydrogels/polymers

**Fig. 12:**
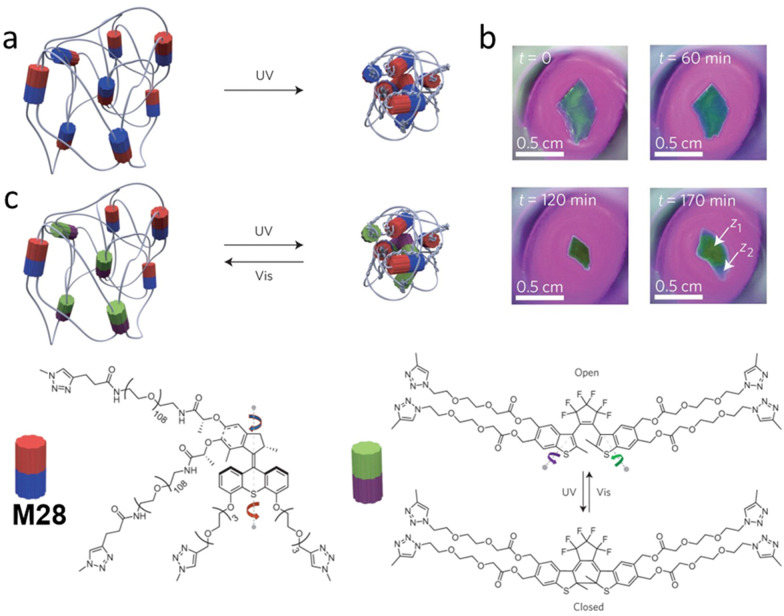
(a) Schematic representation of a reticulated irreversible photo-contractive polymer-motor gel with molecular motor M28 incorporated in the network. (b) Images of contraction of gel *via* UV light irradiation. Reproduced with permission from ref. 41. Copyright 2015, Springer Nature. (c) Schematic representation of a polymer-motor-modulator gel that could be unbraided by a photoswitchable diarylethene modulator. Modified with permission from ref. 101. Copyright 2017, Springer Nature.

Giuseppone and co-workers devised a strategy to utilize continuously unidirectional motion of chiral AMMs to target macroscopic contraction of a polymer hydrogel.^41^ In this study, they first developed a new asymmetric synthesis route that allows for the gram-scale synthesis of enantiopure functional motors.^57^ Subsequently, by using Huisgen [3+2] cycloaddition (‘click reaction’) as a key step, they constructed a polymer hydrogel with motor M28 units reticulated in the network (Fig. 12a). Irradiation with UV light slowly led to an irreversible contraction of the hydrogel volume (up to 80%). This macroscopic shrinkage of a hydrogel (Fig. 12b) confirmed the power of small chiral AMMs to amplify their unidirectional motion from nanoscale to the macroscale. To overcome the shortcoming in this irreversible system, a follow-up paper from the same group described a similar polymer network integrated with an additional diarylethene switching unit, acting as modulators which could unbraid the polymeric chains (Fig. 12c).^101^

### 8.4. Bio-system manipulation

**Fig. 13:**
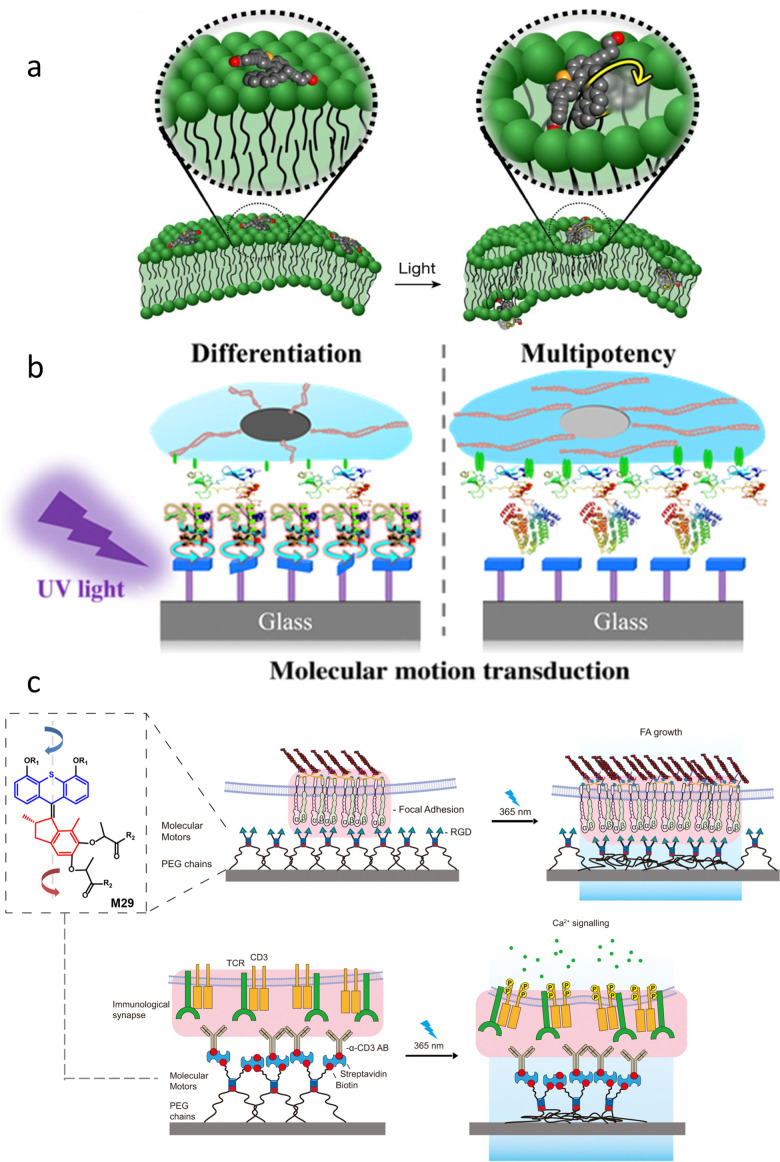
(a) Schematic illustration of a molecular motor atop a cell membrane (left) which can open the membrane by UV light activation of the motor (right). Reproduced with permission from ref. 104. Copyright 2017, Springer Nature. (b) Schematic illustration of a surface assembled rotary motor and the light-induced molecular motion is directing the fate of stem cells.^105^ (c) The design of a force application platform with the rotary motor M29. Schematic representation of manufacture and activation of anti-CD3 antibody linked to the substrate *via* rotatable motor or non-rotatable motor. Reproduced with permission from ref. 106. Copyright 2021, Springer Nature.

Motion is vital in biological systems as dynamic physicochemical and mechanostructural changes determine many bioprocesses.^102^ Inspired by biomolecular machines,^103^ racemic AMMs have been applied recently by Tour and co-workers to perturb and drill into cell membranes *in vitro* using their molecular-scale actuation (Fig. 13a).^104^ Subsequently van Rijn, Feringa and co-workers utilized a different approach to control the communication of AMMs and stem cells at the dynamic interfaces (Fig. 13b).^105^

In another design, Campo and co-workers intercalated polymer chain-linked chiral motor M29 between a membrane receptor and engineered biointerface in order to apply forces by light-triggered contraction of a motorized polymer at cell-matrix and cell–cell junctions (Fig. 13c).^106^ When irradiating at 365 nm, the unidirectional winding of polymeric motor led to the mechanical twisting of the entangled polymer chains, thus effectively “pulling” on engaged cell membrane receptors in the irradiated area. Consequently, the applied forces triggered mechanotransduction processes that promote the force-dependent focal adhesion (FA) maturation and force-dependent T-cell activation.

### 8.5. Control winding/unwinding process

**Fig. 14:**
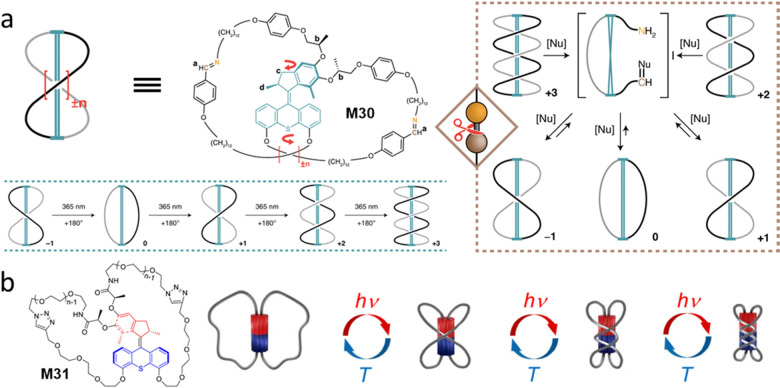
(a) Molecular motor structure M30 and proposed mechanism for the photochemical formation of +3, starting from −1. Thermal relaxation of wound up ±*n* state *via* temporary ring-opening in the presence of catalytic amounts of nucleophile. Reproduced with permission from ref. 107. Copyright 2022, Springer Nature. (b) Molecular whirligig structure M31 and schematic representation of the forward and backward stepwise 180° rotations, leading to new entanglements in the Fig.-of-eight and incorporating 1, 2, or 3 crossings. Reproduced with permission from ref. 108. Copyright 2022, American Chemical Society.

Recently, our group has reported a system in which the AMM can act as a nanoratchet that could shift a coupled chemical equilibrium.^107^ The motor core, M30, is based on a Feringa-type second-generation molecular motor and was synthesized in an enantiomerically pure form to ensure unidirectionality of the system (Fig. 14a). To explore the ability of this AMM to transform work into chemical energy, inspired by dynamic covalent chemistry, a dynamic imine bond was selected to prove this concept. The imine-bridged motorized macrocycle can undergo sequential winding processes to increase the number of entanglements by UV light irradiation, thus populating highly strained topological isomers far from its thermodynamic equilibrium (Fig. 14a). Notably, by adding a catalytic amount of *n*BuNH_2_, the system unwinds *via* imine exchange to re-establish the initial topological equilibrium, which cannot be reversed by thermal relaxation alone (Fig. 14a, right). In this case, the ring-opening reaction of the highly entangled species with *n*BuNH_2_ are irreversible, indicating the high energy of these strained species. These results clearly illustrates that chiral AMMs work as a nanoratchet, capable of driving a coupled chemical equilibrium energetically uphill using light as a power source.

Giuseppone and co-workers reported an independent system that small chiral AMM can act as a light-driven molecular whirligig (Fig. 14b).^108^ In this case, they found the 8-shaped AMM also formed three twisted isomers under light irradiation. Interestingly, by controlling the flexible chain lengths (*n* = 5), the authors found that the reversible untwisting can be converted to its initial state in the dark at 45 °C for 9 d. This finding indicates that in this highly strained system, the inversion of the motor rotation occurred to release the strain in the macrocycle by crossing both thermal helix inversion and *E*/*Z* isomerization barriers at the ground state.

### 8.6. Ion transport

**Fig. 15:**
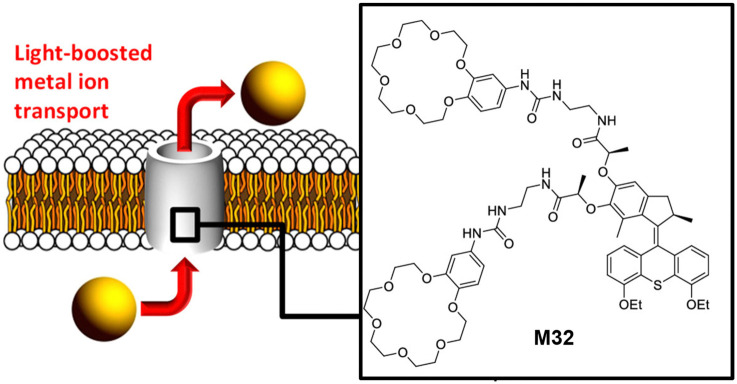
(a) Scheme showing the design of the force light boosted metal ion transport with the rotary motor M32. Reproduced with permission from ref. 109. Copyright 2022, American Chemical Society.

Taking advantage of the ultrafast and unidirectional rotation characteristic of chiral AMMs, the Barboiu and Giuseppone groups reported the use of light-driven AMMs to increase ion transport in phospholipid bilayers.^109^ They demonstrated that out-of-equilibrium actuation dynamics of fast rotary motion leads to the increase of the fractional ion transport activity. The AMM attached with two 18-crown-6 ethers as the macrocyclic units M32 was designed (Fig. 15), due to its high binding affinity to alkali metal ions such as sodium, potassium and rubidium cations. Firstly, control experiments with and without UV irradiation both showed the negligible transport of alkali metal ions in the absence of M32. Interestingly, in the presence of M32 without UV light irradiation, a strong increase of the transport activity was observed M32. Subsequent UV light irradiation led to significant changes of transport activity M32. The highest improvement (400%) was observed for Na^+^ at a concentration of 60 μM. These results suggested the fast unidirectional rotation of chiral AMMs in a continuous out-of-equilibrium manner could boost the ion transport activity through phospholipid bilayers. Recently, the Qu and Bao groups designed a MHz-speed achiral AMM and applied a similar strategy to control K^+^ channel transport in lipid bilayers and induce cancer cell apoptosis.^110^

## 9. Conclusion and outlook

In conclusion, the recent representative examples described in this review have shown that intrinsically chiral light-driven AMMs can act as dynamic chiral small-molecules, opening up a new horizon for emerging applications, with distinct advantages and potential in the future development of nanomachinery and responsive materials. The ability of dynamic chirality and directional (reversible) control over molecular motion with high precision of these AMMs make them undoubtedly unique and highly promising in the field of smart materials. Through the elegant examples, we could draw a picture in the design principle for the choice of AMMs in different applications. Generally, for the multistate-related applications, for instance asymmetric catalysis, the long existence of metastable isomers is crucial for the sufficient chirality transmission, at least being stable during the catalytic process. Under this prerequisite, first generation motors which usually have several hours to days half-life time in (*R*,*R*)-(*M*,*M*)-*cis* state (Fig. 2a) or bistable photoswitches are ideal candidates (Fig. 2d) for this application. In the other hand, for fast dynamic and/or out of equilibrium processes, the second generation motors are privileged molecules to be first taken into consideration owing to their short half lives (Fig. 2c). However, despite the significant progress and well-established design principles in this field, it is still in its very early stages with a number of key challenges that need to be addressed in the future to exploit the full potential of these AMMs with chirality-based functions:

### 1. Red-shifting the absorption wavelength

The operation wavelength of light used in most cases still lies in the UV regime, which limits the applications as well as practical operation of the motors. For instance, highly energetic UV light suffers from limited penetration depth, which will be inefficient when applied into bulk matter, *i.e.* metal organic frameworks (MOFs), covalent organic frameworks (COFs) and porous organic polymers (POPs). UV light can cause harm to sensitive chemical biology systems, and has to be replaced by alternatives such as visible light and near infrared (NIR) light. In addition, AMMs may be susceptible to photo-degradation under UV light irradiation. Therefore, new structures of AMMs with intrinsically red-shifted absorption wavelengths must be developed, or known AMMs must be modified towards operation with visible light or NIR light for instance using sensitizers, 2-photon excitation or upconversion methods.^111^

### 2. Improving photoefficiency

The photoefficiency (*i.e.* the quantum yield of key photoisomerization step) of light-driven AMMs constitutes an ongoing fundamental challenge. Since the development of AMMs in 1999, decades of efforts from our group and many others have led to improve the quantum efficiency of light-driven AMMs. However, this property is more complicated to modify or improve compared to the ground state rotary-speed tuning. There are a few methods that may improve the quantum yields of AMMs, but the functionalization that boost the photo-efficiency may also impede their applicability. Therefore, AMMs with orthogonal accessibility for molecular functioning and high photo-efficiency are highly warranted in the future. Related is the quest for tuning the excited state energy landscape allowing the control over dual functional motors like rotary motion and fluorescence without compromising each function.^112,113^

### 3. High photostationary distributions

High photostationary distributions (PSDs) of AMMs are necessary for specific applications. Considering the control of functions using AMMs as multi-state photoswitches, the high PSD is very important to control dynamic states with high selectivity. In particular, as a switchable asymmetric catalysts or ligands, high PSDs are one of the prerequisites needed to obtain products with high Δe.e. or Δd.r. values. In addition, to precisely control the self-assembly morphology, the high PSD will be useful to understand the self-assembly process in each state (excluding co-assembly of isomers in the PSD). For instance, motor amphiphile M8 (*vide infra*) shows a chiral helical structure of the supramolecular polymer in stable-*cis* state, while showing worm-like fibers in stable-*trans*/metastable-*cis* mixture (32/68) at PSD states.^31^ It is very difficult to distinguish whether the mixture of stable-*trans*/metastable-*cis* mixture or solely the metastable-*cis* isomer itself formed this morphology. Thus, a high PSD in photochemical steps of AMMs is crucial not only to obtain highly enantiopure products based on multistate chiral catalysts, but also to achieve better control in dynamic systems such as chiral supramolecular assemblies.

### 4. As for chirality-led exploration applications, it is essential to obtain sufficient amounts of enantiopure materials of AMMs

In this regard, practical synthetic routes towards chiral AMMs are now developed, but there is still a need for new approaches. Additionally, chiral separation techniques need to be improved, especially for chiral AMMs that cannot yet be synthesized simply and that are difficult to separate in pure enantiomers.

Although many challenges are existing in chiral AMM-based systems, numerous opportunities are open towards chemists to progress these applications one-step at a time. There is still huge potential to explore, as many systems remain elusive:

#### (a) Switchable polymerization in an enantioselective manner

Switchable catalysis to manipulate the microstructures of polymeric materials in a controllable way is appealing and important since the minor changes in tacticity, head-to-tail structure, sequence, and/or chain topology will induce significant effects on the macroscopic properties of a synthetic polymer.^114^ Recently, using photoswitchable catalysts to control polymerization has shown some inspiring advances but remains challenging.^115^ By rational design of chiral motor-based catalysts, there are possibilities to synthesize polymers, which encode chiral information that could be toggled by light.

#### (b) Encoding switchable chiral information in solids for applications

Nowadays emerging porous materials show great advantages in gas and/or guest storage and separation. Incorporation of overcrowded alkene-based molecular motors into porous materials can deliver unidirectional motion^36^ or modulate porosity of the framework materials.^38^ Ideally, by incorporation of enantiopure motor monomers into a solid material framework we could encode chiral information in porous solids, which could be further applied to heterogeneous asymmetric catalysis or switchable enantiomer separation.

#### (c) Photoresponsive supramolecular helical systems

Though supramolecular assembly induced asymmetry transfer and amplification across different length scales is intriguing,^116^ the design of photo-regulated supramolecular helical systems remains challenging.^117^ Therefore, taking advantage of the intrinsically dynamic and reconfigurable chirality of AMMs, there is ample room for the development of reversible supramolecular helical systems based on chiral AMMs.

#### (d) Soft robotics

The bending and helical motion controlled by the chirality of molecular motors in LC networks^94,95^ and supramolecular muscles^42,43^ opens avenues for designing light-responsive artificial soft robotics by programmable construction of AMMs in actuating materials.

#### (e) Orthogonal-/multi-stimuli controlled chirality transfer and delivery systems

To achieve sophisticated functions in responsive systems, it is necessary to use orthogonal-/multi-stimuli to trigger complex tasks in a “smart” way. Such stimuli can control the transmission of chirality in a logic gate system or in a sequential manner, showing high spatiotemporal precision.

#### (f) Responsive surfaces

Incorporation of molecular motors on surfaces renders them able to induce dynamic functions triggered by light. Inspired by the unidirectional molecular motors on surfaces,^33^ in particular the synchronous rotation toward one direction by chiral molecular motors will enable such surfaces to act as mechanical devices or responsive interfaces for specific applications.

#### (g) Biological applications

Molecular motors have been used to open cell membranes by drilling through cellular bilayers,^104^ as well as being applied to control ion transport and the fate of stem cells.^109,110^ While it is still largely unexplored, this field holds bountiful potential for chiral AMMs to achieve chiral recognition in biological systems, such as on–off recognition in distinct chiral states by light-manipulation and control transport and cellular function. Particular appealing are also biohybrid systems, like channel proteins,^118–120^ with artificial motors incorporated at precisely defined positions in there structure to allow non-invasive control.

Light-driven molecular motors with intrinsic chirality embedded in their core open many new avenues for designing responsive and mechanical smart materials and molecular systems with exquisite control over motion at distinct length scales as illustrated in the recent examples discussed here. With the rapid expansion of the AMM family and ongoing interdisciplinary efforts within this field, it is evident that the prospects of light-driven chiral AMMs are bright, lighting up the future of the nanoworld in motion.

## Author contributions

J. S. wrote the initial version of the manuscript and carried out the bibliographic research. D. R. S. P. helped to edit and revise the manuscript. B. L. F. revised and finalized the manuscript and secured funding.

## Conflicts of interest

There are no conflicts to declare.

## Supplementary Material
